# Prevalence of Autism Spectrum Disorder Among Children Aged 8 Years — Autism and Developmental Disabilities Monitoring Network, 11 Sites, United States, 2014

**DOI:** 10.15585/mmwr.ss6706a1

**Published:** 2018-04-27

**Authors:** Jon Baio, Lisa Wiggins, Deborah L. Christensen, Matthew J Maenner, Julie Daniels, Zachary Warren, Margaret Kurzius-Spencer, Walter Zahorodny, Cordelia Robinson Rosenberg, Tiffany White, Maureen S. Durkin, Pamela Imm, Loizos Nikolaou, Marshalyn Yeargin-Allsopp, Li-Ching Lee, Rebecca Harrington, Maya Lopez, Robert T. Fitzgerald, Amy Hewitt, Sydney Pettygrove, John N. Constantino, Alison Vehorn, Josephine Shenouda, Jennifer Hall-Lande, Kim Van Naarden Braun, Nicole F. Dowling

**Affiliations:** ^1^National Center on Birth Defects and Developmental Disabilities, CDC; ^2^University of North Carolina, Chapel Hill; ^3^Vanderbilt University Medical Center, Nashville, Tennessee; ^4^University of Arizona, Tucson; ^5^Rutgers University, Newark, New Jersey; ^6^University of Colorado School of Medicine at the Anschutz Medical Campus; ^7^Colorado Department of Public Health and Environment, Denver; ^8^University of Wisconsin, Madison; ^9^Oak Ridge Institute for Science and Education, Oak Ridge, Tennessee; ^10^Johns Hopkins University, Baltimore, Maryland; ^11^University of Arkansas for Medical Sciences, Little Rock; ^12^Washington University in St. Louis, Missouri; ^13^University of Minnesota, Minneapolis

## Abstract

**Problem/Condition:**

Autism spectrum disorder (ASD).

**Period Covered:**

2014.

**Description of System:**

The Autism and Developmental Disabilities Monitoring (ADDM) Network is an active surveillance system that provides estimates of the prevalence of autism spectrum disorder (ASD) among children aged 8 years whose parents or guardians reside within 11 ADDM sites in the United States (Arizona, Arkansas, Colorado, Georgia, Maryland, Minnesota, Missouri, New Jersey, North Carolina, Tennessee, and Wisconsin). ADDM surveillance is conducted in two phases. The first phase involves review and abstraction of comprehensive evaluations that were completed by professional service providers in the community. Staff completing record review and abstraction receive extensive training and supervision and are evaluated according to strict reliability standards to certify effective initial training, identify ongoing training needs, and ensure adherence to the prescribed methodology. Record review and abstraction occurs in a variety of data sources ranging from general pediatric health clinics to specialized programs serving children with developmental disabilities. In addition, most of the ADDM sites also review records for children who have received special education services in public schools. In the second phase of the study, all abstracted information is reviewed systematically by experienced clinicians to determine ASD case status. A child is considered to meet the surveillance case definition for ASD if he or she displays behaviors, as described on one or more comprehensive evaluations completed by community-based professional providers, consistent with the *Diagnostic and Statistical Manual of Mental Disorders, Fourth Edition, Text Revision* (DSM-IV-TR) diagnostic criteria for autistic disorder; pervasive developmental disorder–not otherwise specified (PDD-NOS, including atypical autism); or Asperger disorder. This report provides updated ASD prevalence estimates for children aged 8 years during the 2014 surveillance year, on the basis of DSM-IV-TR criteria, and describes characteristics of the population of children with ASD. In 2013, the American Psychiatric Association published the *Diagnostic and Statistical Manual of Mental Disorders, Fifth Edition* (DSM-5), which made considerable changes to ASD diagnostic criteria. The change in ASD diagnostic criteria might influence ADDM ASD prevalence estimates; therefore, most (85%) of the records used to determine prevalence estimates based on DSM-IV-TR criteria underwent additional review under a newly operationalized surveillance case definition for ASD consistent with the DSM-5 diagnostic criteria. Children meeting this new surveillance case definition could qualify on the basis of one or both of the following criteria, as documented in abstracted comprehensive evaluations: 1) behaviors consistent with the DSM-5 diagnostic features; and/or 2) an ASD diagnosis, whether based on DSM-IV-TR or DSM-5 diagnostic criteria. Stratified comparisons of the number of children meeting either of these two case definitions also are reported.

**Results:**

For 2014, the overall prevalence of ASD among the 11 ADDM sites was 16.8 per 1,000 (one in 59) children aged 8 years. Overall ASD prevalence estimates varied among sites, from 13.1–29.3 per 1,000 children aged 8 years. ASD prevalence estimates also varied by sex and race/ethnicity. Males were four times more likely than females to be identified with ASD. Prevalence estimates were higher for non-Hispanic white (henceforth, white) children compared with non-Hispanic black (henceforth, black) children, and both groups were more likely to be identified with ASD compared with Hispanic children. Among the nine sites with sufficient data on intellectual ability, 31% of children with ASD were classified in the range of intellectual disability (intelligence quotient [IQ] <70), 25% were in the borderline range (IQ 71–85), and 44% had IQ scores in the average to above average range (i.e., IQ >85). The distribution of intellectual ability varied by sex and race/ethnicity. Although mention of developmental concerns by age 36 months was documented for 85% of children with ASD, only 42% had a comprehensive evaluation on record by age 36 months. The median age of earliest known ASD diagnosis was 52 months and did not differ significantly by sex or race/ethnicity. For the targeted comparison of DSM-IV-TR and DSM-5 results, the number and characteristics of children meeting the newly operationalized DSM-5 case definition for ASD were similar to those meeting the DSM-IV-TR case definition, with DSM-IV-TR case counts exceeding DSM-5 counts by less than 5% and approximately 86% overlap between the two case definitions (kappa = 0.85).

**Interpretation:**

Findings from the ADDM Network, on the basis of 2014 data reported from 11 sites, provide updated population-based estimates of the prevalence of ASD among children aged 8 years in multiple communities in the United States. The overall ASD prevalence estimate of 16.8 per 1,000 children aged 8 years in 2014 is higher than previously reported estimates from the ADDM Network. Because the ADDM sites do not provide a representative sample of the entire United States, the combined prevalence estimates presented in this report cannot be generalized to all children aged 8 years in the United States. Consistent with reports from previous ADDM surveillance years, findings from 2014 were marked by variation in ASD prevalence when stratified by geographic area, sex, and level of intellectual ability. Differences in prevalence estimates between black and white children have diminished in most sites, but remained notable for Hispanic children. For 2014, results from application of the DSM-IV-TR and DSM-5 case definitions were similar, overall and when stratified by sex, race/ethnicity, DSM-IV-TR diagnostic subtype, or level of intellectual ability.

**Public Health Action:**

Beginning with surveillance year 2016, the DSM-5 case definition will serve as the basis for ADDM estimates of ASD prevalence in future surveillance reports. Although the DSM-IV-TR case definition will eventually be phased out, it will be applied in a limited geographic area to offer additional data for comparison. Future analyses will examine trends in the continued use of DSM-IV-TR diagnoses, such as autistic disorder, PDD-NOS, and Asperger disorder in health and education records, documentation of symptoms consistent with DSM-5 terminology, and how these trends might influence estimates of ASD prevalence over time. The latest findings from the ADDM Network provide evidence that the prevalence of ASD is higher than previously reported estimates and continues to vary among certain racial/ethnic groups and communities. With prevalence of ASD ranging from 13.1 to 29.3 per 1,000 children aged 8 years in different communities throughout the United States, the need for behavioral, educational, residential, and occupational services remains high, as does the need for increased research on both genetic and nongenetic risk factors for ASD.

## Introduction

Autism spectrum disorder (ASD) is a developmental disability defined by diagnostic criteria that include deficits in social communication and social interaction, and the presence of restricted, repetitive patterns of behavior, interests, or activities that can persist throughout life (*1*). CDC began tracking the prevalence of ASD and characteristics of children with ASD in the United States in 1998 (*2*,*3*). The first CDC study, which was based on an investigation in Brick Township, New Jersey (*2*), identified similar characteristics but higher prevalence of ASD compared with other studies of that era. The second CDC study, which was conducted in metropolitan Atlanta, Georgia (*3*), identified a lower prevalence of ASD compared with the Brick Township study but similar estimates compared with other prevalence studies of that era. In 2000, CDC established the Autism and Developmental Disabilities Monitoring (ADDM) Network to collect data that would provide estimates of the prevalence of ASD and other developmental disabilities in the United States (*4*,*5*).

Tracking the prevalence of ASD poses unique challenges because of the heterogeneity in symptom presentation, lack of biologic diagnostic markers, and changing diagnostic criteria (*5*). Initial signs and symptoms typically are apparent in the early developmental period; however, social deficits and behavioral patterns might not be recognized as symptoms of ASD until a child is unable to meet social, educational, occupational, or other important life stage demands (*1*). Features of ASD might overlap with or be difficult to distinguish from those of other psychiatric disorders, as described extensively in DSM-5 (*1*). Although standard diagnostic tools have been validated to inform clinicians’ impressions of ASD symptomology, inherent complexity of measurement approaches and variation in clinical impressions and decision-making, combined with policy changes that affect eligibility for health benefits and educational programs, complicates identification of ASD as a behavioral health diagnosis or educational exceptionality. To reduce the influence of these factors on prevalence estimates, the ADDM Network has consistently tracked ASD by applying a surveillance case definition of ASD and using the same record-review methodology and behaviorally defined case inclusion criteria since 2000 (*5*).

ADDM estimates of ASD prevalence among children aged 8 years in multiple U.S. communities have increased from approximately one in 150 children during 2000–2002 to one in 68 during 2010–2012, more than doubling during this period (*6*–*11*). The observed increase in ASD prevalence underscores the need for continued surveillance using consistent methods to monitor the changing prevalence of ASD and characteristics of children with ASD in the population.

In addition to serving as a basis for ASD prevalence estimates, ADDM data have been used to describe characteristics of children with ASD in the population, to study how these characteristics vary with ASD prevalence estimates over time and among communities, and to monitor progress toward *Healthy People 2020* objectives (*12*). ADDM ASD prevalence estimates consistently estimated a ratio of approximately 4.5 male:1 female with ASD during 2006–2012 (*9*–*11*). Other characteristics that have remained relatively constant over time in the population of children identified with ASD by ADDM include the median age of earliest known ASD diagnosis, which remained close to 53 months during 2000–2012 (range: 50 months [2012] to 56 months [2002]), and the proportion of children receiving a comprehensive developmental evaluation by age 3 years, which remained close to 43% during 2006–2012 (range: 43% [2006 and 2012] to 46% [2008]).

ASD prevalence by race/ethnicity has been more varied over time among ADDM Network communities (*9*–*11*). Although ASD prevalence estimates have historically been greater among white children compared with black or Hispanic children (*13*), ADDM-reported white:black and white:Hispanic prevalence ratios have declined over time because of larger increases in ASD prevalence among black children and, to an even greater extent, among Hispanic children, as compared with the magnitude of increase in ASD prevalence among white children (*9*). Previous reports from the ADDM Network estimated ASD prevalence among white children to exceed that among black children by approximately 30% in 2002, 2006, and 2010, and by approximately 20% in 2008 and 2012. Estimated prevalence among white children exceeded that among Hispanic children by nearly 70% in 2002 and 2006, and by approximately 50% in 2008, 2010, and 2012. ASD prevalence estimates from the ADDM Network also have varied by socioeconomic status (SES). A consistent pattern observed in ADDM data has been higher identified ASD prevalence among residents of neighborhoods with higher socioeconomic status (SES). Although ASD prevalence has increased over time at all levels of SES, the absolute difference in prevalence between high, middle, and lower SES did not change from 2002 to 2010 (*14*,*15*). In the context of declining white:black and white:Hispanic prevalence ratios amidst consistent SES patterns, a complex three-way interaction among time, SES, and race/ethnicity has been proposed (*16*).

Finally, ADDM Network data have shown a shift toward children with ASD with higher intellectual ability (*9*–*11*), as the proportion of children with ASD whose intelligence quotient (IQ) scores fell within the range of intellectual disability (ID) (i.e., IQ <70) has decreased gradually over time. During 2000–2002, approximately half of children with ASD had IQ scores in the range of ID; during 2006–2008, this proportion was closer to 40%; and during 2010–2012, less than one third of children with ASD had IQ ≤70 (*9*–*11*). This trend was more pronounced for females as compared with males (*9*). The proportion of males with ASD and ID declined from approximately 40% during 2000–2008 (*9*) to 30% during 2010–2012 (*10*,*11*). The proportion of females with ASD and ID declined from approximately 60% during 2000–2002, to 45% during 2006–2008, and to 35% during 2010–2012 (*9*–*11*).

All previously reported ASD prevalence estimates from the ADDM Network were based on a surveillance case definition aligned with DSM-IV-TR diagnostic criteria for autistic disorder; pervasive developmental disorder–not otherwise specified (PDD-NOS, including atypical autism); or Asperger disorder. In the American Psychiatric Association’s 2013 publication of DSM-5, substantial changes were made to the taxonomy and diagnostic criteria for autism (*1*,*17*). Taxonomy changed from Pervasive Developmental Disorders, which included multiple diagnostic subtypes, to autism spectrum disorder, which no longer comprises distinct subtypes but represents one singular diagnostic category defined by level of support needed by the individual. Diagnostic criteria were refined by collapsing the DSM-IV-TR social and communication domains into a single, combined domain for DSM-5. Persons diagnosed with ASD under DSM-5 must meet all three criteria under the social communication/interaction domain (i.e., deficits in social-emotional reciprocity; deficits in nonverbal communicative behaviors; and deficits in developing, understanding, and maintaining relationships) and at least two of the four criteria under the restrictive/repetitive behavior domain (i.e., repetitive speech or motor movements, insistence on sameness, restricted interests, or unusual response to sensory input).

Although the DSM-IV-TR criteria proved useful in identifying ASD in some children, clinical agreement and diagnostic specificity in some subtypes (e.g., PDD-NOS) was poor, offering empirical support to the notion of two, rather than three, diagnostic domains. The DSM-5 introduced a framework to address these concerns (*18*), while maintaining that any person with an established DSM-IV-TR diagnosis of autistic disorder, Asperger disorder, or PDD-NOS would automatically qualify for a DSM-5 diagnosis of autism spectrum disorder. Previous studies suggest that DSM-5 criteria for ASD might exclude certain children who would have qualified for a DSM-IV-TR diagnosis but had not yet received one, particularly those who are very young and those without ID (*19*–*23*). These findings suggest that ASD prevalence estimates will likely be lower under DSM-5 than they have been under DSM-IV-TR diagnostic criteria.

This report provides the latest available ASD prevalence estimates from the ADDM Network based on both DSM-IV-TR and DSM-5 criteria and asserts the need for future monitoring of ASD prevalence trends and efforts to improve early identification of ASD. The intended audiences for these findings include pediatric health care providers, school psychologists, educators, researchers, policymakers, and program administrators working to understand and address the needs of persons with ASD and their families. These data can be used to help plan services, guide research into risk factors and effective interventions, and inform policies that promote improved outcomes in health and education settings.

## Methods

### Study Sites

The Children’s Health Act (*4*) authorized CDC to monitor prevalence of ASD in multiple areas of the United States, a charge that led to the formation of the ADDM Network in 2000. Since that time, CDC has funded grantees in 16 states (Alabama, Arizona, Arkansas, Colorado, Florida, Maryland, Minnesota, Missouri, New Jersey, North Carolina, Pennsylvania, South Carolina, Tennessee, Utah, West Virginia, and Wisconsin). CDC tracks ASD in metropolitan Atlanta and represents the Georgia site collaborating with competitively funded sites to form the ADDM Network.

The ADDM Network uses multisite, multisource, records-based surveillance based on a model originally implemented by CDC’s Metropolitan Atlanta Developmental Disabilities Surveillance Program (MADDSP) (*24*). As feasible, the surveillance methods have remained consistent over time. Certain minor changes have been introduced to improve efficiency and data quality. Although a different array of geographic areas was covered in each of the eight biennial ADDM Network surveillance years spanning 2000–2014, these changes have been documented to facilitate evaluation of their impact.

The core surveillance activities in all ADDM Network sites focus on children aged 8 years because the baseline ASD prevalence study conducted by MADDSP suggested that this is the age of peak prevalence (*3*). ADDM has multiple goals: 1) to provide descriptive data on classification and functioning of the population of children with ASD, 2) to monitor the prevalence of ASD in different areas of the United States, and 3) to understand the impact of ASD in U.S. communities.

Funding for ADDM Network sites participating in the 2014 surveillance year was awarded for a 4-year cycle covering 2015–2018, during which time data were collected for children aged 8 years during 2014 and 2016. Sites were selected through a competitive objective review process on the basis of their ability to conduct active, records-based surveillance of ASD; they were not selected to be a nationally representative sample. A total of 11 sites are included in the current report (Arizona, Arkansas, Colorado, Georgia, Maryland, Minnesota, Missouri, New Jersey, North Carolina, Tennessee, and Wisconsin). Each ADDM site participating in the 2014 surveillance year functioned as a public health authority under the Health Insurance Portability and Accountability Act of 1996 Privacy Rule and met applicable local Institutional Review Board and privacy and confidentiality requirements under 45 CFR 46 (*25*).

### Case Ascertainment

ADDM is an active surveillance system that does not depend on family or practitioner reporting of an existing ASD diagnosis or classification to determine ASD case status. ADDM staff conduct surveillance to determine case status in a two-phase process. The first phase of ADDM involves review and abstraction of children’s evaluation records from data sources in the community. In the second phase, all abstracted evaluations for each child are compiled in chronological order into a comprehensive record that is reviewed by one or more experienced clinicians to determine the child’s ASD case status. Developmental assessments completed by a wide range of health and education providers are reviewed. Data sources are categorized as either 1) education source type, including evaluations to determine eligibility for special education services or 2) health source type, including diagnostic and developmental assessments from psychologists, neurologists, developmental pediatricians, child psychiatrists, physical therapists, occupational therapists, and speech/language pathologists. Agreements to access records are made at the institutional level in the form of contracts, memoranda, or other formal agreements.

All ADDM Network sites have agreements in place to access records at health sources; however, despite the otherwise standardized approach, not all sites have permission to access education records. One ADDM site (Missouri) has not been granted access to records at any education sources. Among the remaining sites, some receive permission from their statewide Department of Education to access children’s educational records, whereas other sites must negotiate permission from numerous individual school districts to access educational records. Six sites (Arizona, Georgia, Maryland, Minnesota, New Jersey, and North Carolina) reviewed education records for all school districts in their covered surveillance areas. Three ADDM sites (Colorado, Tennessee, and Wisconsin) received permission to review education records in only certain school districts within the overall geographic area covered for 2014. In Tennessee, permission to access education records was granted from 13 of 14 school districts in the 11-county surveillance area, representing 88% of the total population of children aged 8 years. Conversely, access to education records was limited to a small proportion of the population in the overall geographic area covered by two sites (33% in Colorado and 26% in Wisconsin). In the Colorado school districts where access to education records is permitted for ADDM, parents are directly notified about the ADDM system and can request that their children’s education records be excluded. The Arkansas ADDM site received permission from their state Department of Education to access children’s educational records statewide; however, time and travel constraints prevented investigators from visiting all 250 school districts in the 75-county surveillance area, resulting in access to education records for 69% of the statewide population of children aged 8 years. The two sites with access to education records throughout most, but not all, of the surveillance area (Arkansas and Tennessee) received data from their state Department of Education to evaluate the potential impact on reported ASD prevalence estimates attributed to missing records.

Within each education and health data source, ADDM sites identify records to review based on a child’s year of birth and one or more selected eligibility classifications for special education or *International Classification of Diseases, Ninth Revision* (ICD-9) billing codes for select childhood disabilities or psychological conditions. Children’s records are first reviewed to confirm year of birth and residency in the surveillance area at some time during the surveillance year. For children meeting these requirements, the records are then reviewed for certain behavioral or diagnostic descriptions defined by ADDM as triggers for abstraction (e.g., child does not initiate interactions with others, prefers to play alone or engage in solitary activities, or has received a documented ASD diagnosis). If abstraction triggers are found, evaluation information from birth through the current surveillance year from all available sources is abstracted into a single composite record for each child.

In the second phase of surveillance, the abstracted composite evaluation files are deidentified and reviewed systematically by experienced clinicians who have undergone standardized training to determine ASD case status using a coding scheme based on the DSM-IV-TR guidelines. A child meets the surveillance case definition for ASD if behaviors described in the composite record are consistent with the DSM-IV-TR diagnostic criteria for any of the following conditions: autistic disorder, PDD-NOS (including atypical autism), or Asperger disorder (Box 1). A child might be disqualified from meeting the surveillance case definition for ASD if, based on the clinical judgment of one or more reviewers, there is insufficient or conflicting information in support of ASD, sufficient information to rule out ASD, or if one or more other diagnosed conditions better account for the child’s symptoms.

BOX 1 **Autism spectrum disorder (ASD) case determination criteria under DSM-IV-TR**

**Table:** 

DSM-IV-TR behavioral criteria	
**Social**	**1a. Marked impairment in the use of multiple nonverbal behaviors, such as eye-to-eye gaze, facial expression, body postures, and gestures to regulate social interaction** **1b. Failure to develop peer relationships appropriate to developmental level** **1c. A lack of spontaneous seeking to share enjoyment, interests, or achievements with other people (e.g., by a lack of showing, bringing, or pointing out objects of interest)** **1d. Lack of social or emotional reciprocity**
**Communication**	**2a. Delay in, or total lack of, the development of spoken language (not accompanied by an attempt to compensate through alternative modes of communication, such as gesture or mime)** **2b. In individuals with adequate speech, marked impairment in the ability to initiate or sustain a conversation with others** **2c. Stereotyped and repetitive use of language or idiosyncratic language** **2d. Lack of varied, spontaneous make-believe play or social imitative play appropriate to developmental level**
**Restricted behavior/Interest**	**3a. Encompassing preoccupation with one or more stereotyped and restricted patterns of interest that is abnormal either in intensity or focus** **3b. Apparently inflexible adherence to specific, nonfunctional routines, or rituals** **3c. Stereotyped and repetitive motor mannerisms (e.g., hand or finger flapping or twisting, or complex whole body movements)** **3d. Persistent preoccupation with parts of objects**
**Developmental history**	**Child had identified delays or any concern with development in the following areas at or before the age of 3 years: Social, Communication, Behavior, Play, Motor, Attention, Adaptive, Cognitive**
**Autism discriminators**	**Oblivious to children** **Oblivious to adults or others** **Rarely responds to familiar social approach** **Language primarily echolalia or jargon** **Regression/loss of social, language, or play skills** **Previous ASD diagnosis, whether based on DSM-IV-TR or DSM-5 diagnostic criteria** **Lack of showing, bringing, etc.** **Little or no interest in others** **Uses others as tools** **Repeats extensive dialog** **Absent or impaired imaginative play** **Markedly restricted interests** **Unusual preoccupation** **Insists on sameness** **Nonfunctional routines** **Excessive focus on parts** **Visual inspection** **Movement preoccupation** **Sensory preoccupation**
**DSM-IV-TR case determination**	**At least six behaviors coded with a minimum of two Social, one Communication, and one Restricted Behavior/Interest; AND evidence of developmental delay or concern at or before the age of 3 years** **OR** **At least two behaviors coded with a minimum of one Social and either one Communication and/or one Restricted Behavior/Interest; AND at least one autism discriminator coded** **Note: A child might be disqualified from meeting the DSM-IV-TR surveillance case definition for ASD if, based on the clinical judgment of one or more reviewers, there is insufficient or conflicting information in support of ASD, sufficient information to rule out ASD, or if one or more other diagnosed conditions better account for the child’s symptoms**

**Abbreviation:** DSM-IV-TR = *Diagnostic and Statistical Manual of Mental Disorders, Fourth Edition (Text Revision).*

Although new diagnostic criteria became available in 2013, the children under surveillance in 2014 would have grown up primarily under the DSM-IV-TR definitions for ASD, which are prioritized in this report. The 2014 surveillance year is the first to operationalize an ASD case definition based on DSM-5 diagnostic criteria, in addition to that based on DSM-IV-TR. Because of delays in developing information technology systems to manage data collected under this new case definition, the surveillance area for DSM-5 was reduced by 19% in an effort to include complete estimates for both DSM-IV-TR and DSM-5 in this report. Phase 1 record review and abstraction was the same for DSM-IV-TR and DSM-5; however, a coding scheme based on the DSM-5 definition of ASD was developed for Phase 2 of the ADDM methodology (i.e., systematic review by experienced clinicians). The new coding scheme was developed through a collaborative process and includes reliability measures, although no validation metrics have been published for this new ADDM Network DSM-5 case definition. A child could meet the DSM-5 surveillance case definition for ASD under one or both of the following criteria, as documented in abstracted comprehensive evaluations: 1) behaviors consistent with the DSM-5 diagnostic features; and/or 2) an ASD diagnosis, whether based on DSM-IV-TR or DSM-5 diagnostic criteria (Box 2). Children with a documented ASD diagnosis were included as meeting the DSM-5 surveillance case definition for two reasons. First, published DSM-5 diagnostic criteria include the presence of a DSM-IV-TR diagnosis of autistic disorder, PDD-NOS, or Asperger disorder, to ensure continuity of diagnoses and services. Second, sensitivity of the DSM-5 surveillance case definition might be increased when counting children diagnosed with ASD by a qualified professional, based on either DSM-IV-TR or DSM-5 criteria, whether or not all DSM-5 social and behavioral criteria are documented in abstracted comprehensive evaluations. The ADDM Network methods allow differentiation of those meeting the surveillance case status based on one or both criteria. Consistent with the DSM-IV-TR case definition, a child might be disqualified from meeting the DSM-5 surveillance case definition for ASD if, based on the clinical judgment of one or more reviewers, there is insufficient or conflicting information in support of ASD, sufficient information to rule out ASD, or if one or more other diagnosed conditions better account for the child’s symptoms. In this report, prevalence estimates are based on the DSM-IV-TR case definition, whereas case counts are presented and compared for children meeting the DSM-IV-TR and/or DSM-5 case definitions.

BOX 2 **Autism spectrum disorder case determination criteria under DSM-5**

**Table:** 

DSM-5 behavioral criteria	
A. Persistent deficits in social communication and social interaction	A1: Deficits in social emotional reciprocity A2. Deficits in nonverbal communicative behaviors A3. Deficits in developing, maintaining, and understanding relationships
B. Restricted, repetitive patterns of behavior, interests, or activities, currently or by history	B1: Stereotyped or repetitive motor movements, use of objects or speech B2. Insistence on sameness, inflexible adherence to routines, or ritualized patterns of verbal or nonverbal behavior B3. Highly restricted interests that are abnormal in intensity or focus B4. Hyper- or hypo-reactivity to sensory input or unusual interest in sensory aspects of the environment
**Historical PDD diagnosis**	Any ASD diagnosis documented in a comprehensive evaluation, including a DSM-IV diagnosis of autistic disorder, Asperger disorder, or pervasive developmental disorder–not otherwise specified (PDD-NOS)
**DSM-5 case determination**	All three behavioral criteria coded under part A, and at least two behavioral criteria coded under part B OR Any ASD diagnosis documented in a comprehensive evaluation, whether based on DSM-IV-TR or DSM-5 diagnostic criteria Note: A child might be disqualified from meeting the DSM-5 surveillance case definition for ASD if, based on the clinical judgment of one or more reviewers, there is insufficient or conflicting information in support of ASD, sufficient information to rule out ASD, or if one or more other diagnosed conditions better account for the child’s symptoms

**Abbreviation:** DSM-5 = *Diagnostic and Statistical Manual of Mental Disorders, Fifth Edition*.

### Quality Assurance

All sites follow the quality assurance standards established by the ADDM Network. In the first phase, the accuracy of record review and abstraction is checked periodically. In the second phase, interrater reliability is monitored on an ongoing basis using a blinded, random 10% sample of abstracted records that are scored independently by two reviewers (*5*). For 2014, interrater agreement on DSM-IV-TR case status (confirmed ASD versus not ASD) was 89.1% when comparison samples from all sites were combined (k = 0.77), which was slightly below quality assurance standards established for the ADDM Network (90% agreement, 0.80 kappa). On DSM-5 reviews, interrater agreement on case status (confirmed ASD versus not ASD) was 92.3% when comparison samples from all sites were combined (k = 0.84). Thus, for the DSM-5 surveillance definition, reliability exceeded quality assurance standards established for the ADDM Network.

#### Descriptive Characteristics and Data Sources

Each ADDM site attempted to obtain birth certificate data for all children abstracted during Phase 1 through linkages conducted using state vital records. These data were only available for children born in the state where the ADDM site is located. The race/ethnicity of each child was determined from information contained in source records or, if not found in the source file, from birth certificate data on one or both parents. Children with race coded as “other” or “multiracial” were considered to be missing race information for all analyses that were stratified by race/ethnicity. For this report, data on timing of the first comprehensive evaluation on record were restricted to children with ASD who were born in the state where the ADDM site is located, as confirmed by linkage to birth certificate records. Data were restricted in this manner to reduce errors in the estimate that were introduced by children for whom evaluation records were incomplete because they were born out of state and migrated into the surveillance area between the time of birth and the year when they reached age 8 years.

Information on children’s functional skills is abstracted from source records when available, including scores on tests of adaptive behavior and intellectual ability. Because no standardized, validated measures of functioning specific to ASD have been widely adopted in clinical practice and because adaptive behavior rating scales are not sufficiently available in health and education records of children with ASD, scores of intellectual ability have remained the primary source of information on children’s functional skills. Children are classified as having ID if they have an IQ score of ≤70 on their most recent test available in the record. Borderline intellectual ability is defined as having an IQ score of 71–85, and average or above-average intellectual ability is defined as having an IQ score of >85. In the absence of a specific IQ score, an examiner’s statement based on a formal assessment of the child’s intellectual ability, if available, is used to classify the child in one of these three levels.

Diagnostic conclusions from each evaluation record are summarized for each child, including notation of any ASD diagnosis by subtype, when available. Children are considered to have a previously documented ASD classification if they received a diagnosis of autistic disorder, PDD-NOS, Asperger disorder, or ASD that was documented in an abstracted evaluation or by an ICD-9 billing code at any time from birth through the year when they reached age 8 years, or if they were noted as meeting eligibility criteria for special education services under the classification of autism or ASD.

#### Analytic Methods

Population denominators for calculating ASD prevalence estimates were obtained from the National Center for Health Statistics Vintage 2016 Bridged-Race Postcensal Population Estimates (*26*). CDC’s National Vital Statistics System provides estimated population counts by state, county, single year of age, race, ethnic origin, and sex. Population denominators for the 2014 surveillance year were compiled from postcensal estimates of the number of children aged 8 years living in the counties under surveillance by each ADDM site (Table 1).

**TABLE 1:** Number* and percentage of children aged 8 years, by race/ethnicity and site — Autism and Developmental Disabilities Monitoring Network, 11 sites, United States, 2014

Site	Site institution	Surveillance area	Total	White, non-Hispanic		Black, non-Hispanic		Hispanic		Asian or Pacific Islander, non-Hispanic		American Indian or Alaska Native, non-Hispanic	
			No.	No.	(%)	No.	(%)	No.	(%)	No.	(%)	No.	(%)
Arizona	University of Arizona	Part of 1 county in metropolitan Phoenix^†^	**24,952**	12,308	(49.3)	1,336	(5.4)	9,792	(39.2)	975	(3.9)	541	(2.2)
Arkansas	University of Arkansas for Medical Sciences	All 75 counties in Arkansas	**39,992**	26,103	(65.3)	7,705	(19.3)	5,012	(12.5)	843	(2.1)	329	(0.8)
Colorado	Colorado Department of Public Health and Environment	7 counties in metropolitan Denver	**41,128**	22,410	(54.5)	2,724	(6.6)	13,735	(33.4)	2,031	(4.9)	228	(0.6)
Georgia	CDC	5 counties including metropolitan Atlanta	**51,161**	15,495	(30.3)	22,042	(43.1)	9,913	(19.4)	3,599	(7.0)	112	(0.2)
Maryland	Johns Hopkins University	1 county in metropolitan Baltimore	**9,955**	4,977	(50.0)	3,399	(34.1)	829	(8.3)	719	(7.2)	31	(0.3)
Minnesota	University of Minnesota	Parts of 2 counties including Minneapolis–St. Paul^†^	**9,767**	3,793	(38.8)	2,719	(27.8)	1,486	(15.2)	1,576	(16.1)	193	(2.0)
Missouri	Washington University	5 counties including metropolitan St. Louis	**25,333**	16,529	(65.2)	6,577	(26.0)	1,220	(4.8)	931	(3.7)	76	(0.3)
New Jersey	Rutgers University	4 counties including metropolitan Newark	**32,935**	13,593	(41.3)	7,166	(21.8)	10,226	(31.0)	1,874	(5.7)	76	(0.2)
North Carolina	University of North Carolina–Chapel Hill	6 counties in central North Carolina	**30,283**	15,241	(50.3)	7,701	(25.4)	5,463	(18.0)	1,778	(5.9)	100	(0.3)
Tennessee	Vanderbilt University Medical Center	11 counties in middle Tennessee	**24,940**	15,867	(63.6)	4,896	(19.6)	3,324	(13.3)	799	(3.2)	54	(0.2)
Wisconsin	University of Wisconsin–Madison	10 counties in southeastern Wisconsin	**35,037**	20,732	(59.2)	6,486	(18.5)	6,181	(17.6)	1,471	(4.2)	167	(0.5)
**All sites combined**			**325,483**	**167,048**	**(51.3)**	**72,751**	**(22.4)**	**67,181**	**(20.6)**	**16,596**	**(5.1)**	**1,907**	**(0.6)**

* Total numbers of children aged 8 years in each surveillance area were obtained from CDC’s National Center for Health Statistics Vintage 2016 Bridged-Race Population Estimates for July 1, 2014.

^†^ Denominator excludes school districts that were not included in the surveillance area, calculated from National Center for Education Statistics enrollment counts of third graders during the 2014–2015 school year.

In two sites (Arizona and Minnesota), geographic boundaries were defined by constituent school districts included in the surveillance area. The number of children living in outlying school districts was subtracted from the county-level census denominators using school enrollment data from the U.S. Department of Education’s National Center for Education Statistics (*27*). Enrollment counts of students in third grade during the 2014–15 school year differed from the CDC bridged-race population estimates, attributable primarily to children being enrolled out of the customary grade for their age or in charter schools, home schools, or private schools. Because these differences varied by race and sex within the applicable counties, race- and sex-specific adjustments based on enrollment counts were applied to the CDC population estimates to derive school district-specific denominators for Arizona and Minnesota.

Race- or ethnicity-specific prevalence estimates were calculated for four groups: white, black, Hispanic (regardless of race), and Asian/Pacific Islander. Prevalence results are reported as the total number of children meeting the ASD case definition per 1,000 children aged 8 years in the population in each race/ethnicity group. ASD prevalence also was estimated separately for boys and girls and within each level of intellectual ability. Overall prevalence estimates include all children identified with ASD regardless of sex, race/ethnicity, or level of intellectual ability and thus are not affected by the availability of data on these characteristics.

Statistical tests were selected and confidence intervals (CIs) for prevalence estimates were calculated under the assumption that the observed counts of children identified with ASD were obtained from an underlying Poisson distribution with an asymptotic approximation to the normal. Pearson chi-square tests were performed, and prevalence ratios and percentage differences were calculated to compare prevalence estimates from different strata. Kappa statistics were computed to describe concordance between the DSM-IV-TR and DSM-5 case definitions, as well as to describe interrater agreement on either case definition for quality assurance. Pearson chi-square tests also were performed for testing significance in comparisons of proportions, and unadjusted odds ratio (OR) estimates were calculated to further describe these comparisons. In an effort to reduce the effect of outliers, distribution medians were typically presented, although one-way ANOVA was used to test significance when comparing arithmetic means of these distributions. Significance was set at p<0.05. Results for all sites combined were based on pooled numerator and denominator data from all sites, in total and stratified by race/ethnicity, sex, and level of intellectual ability.

#### Sensitivity Analysis Methods

Certain education and health records were missing for certain children, including records that could not be located for review, those affected by the passive consent process unique to the Colorado site, and those archived and deemed too costly to retrieve. A sensitivity analysis of the effect of these missing records on case ascertainment was conducted. All children initially identified for record review were first stratified by two factors closely associated with final case status: information source (health source type only, education source type only, or both source types) and the presence or absence of either an autism special education eligibility or an ICD-9-CM code for ASD, collectively forming six strata. The potential number of cases not identified because of missing records was estimated under the assumption that within each of the six strata, the proportion of children confirmed as ASD surveillance cases among those with missing records would be similar to the proportion of cases among children with no missing records. Within each stratum, the proportion of children with no missing records who were confirmed as having ASD was applied to the number of children with missing records to estimate the number of missed cases, and the estimates from all six strata were added to calculate the total for each site. This sensitivity analysis was conducted solely to investigate the potential impact of missing records on the presented estimates. The estimates presented in this report do not reflect this adjustment or any of the other assessments of the potential effects of assumptions underlying the approach.

All ADDM sites identified records for review from health sources by conducting record searches that were based on a common list of ICD-9 billing codes. Because several sites were conducting surveillance for other developmental disabilities in addition to ASD (i.e., one or more of the following: cerebral palsy, ID, hearing loss, and vision impairment), they reviewed records based on an expanded list of ICD-9 codes. The Colorado site also requested code 781.3 (lack of coordination), which was identified in that community as a commonly used billing code for children with ASD. The proportion of children meeting the ASD surveillance case definition whose records were obtained solely on the basis of those additional codes was calculated to evaluate the potential impact on ASD prevalence.

## Results

A total population of 325,483 children aged 8 years was covered by the 11 ADDM sites that provided data for the 2014 surveillance year (Table 1). This number represented 8% of the total U.S. population of children aged 8 years in 2014 (4,119,668) (*19*). A total of 53,120 records for 42,644 children were reviewed from health and education sources. Of these, the source records of 10,886 children met the criteria for abstraction, which was 25.5% of the total number of children whose source records were reviewed and 3.3% of the population under surveillance. Of the records reviewed by clinicians, 5,473 children met the ASD surveillance case definition. The number of evaluations abstracted for each child who was ultimately identified with ASD varied by site (median: five; range: three [Arizona, Minnesota, Missouri, and Tennessee] to 10 [Maryland]).

### Overall ASD Prevalence Estimates

Overall ASD prevalence for the ADDM 2014 surveillance year varied widely among sites (range: 13.1 [Arkansas] to 29.3 [New Jersey]) (Table 2). On the basis of combined data from all 11 sites, ASD prevalence was 16.8 per 1,000 (one in 59) children aged 8 years. Overall estimated prevalence of ASD was highest in New Jersey (29.3) compared to each of the other ten sites (p<0.01).

**TABLE 2:** Estimated prevalence* of autism spectrum disorder among children aged 8 years, by sex — Autism and Developmental Disabilities Monitoring Network, 11 sites, United States, 2014

Site	Total population	Total no. with ASD	Sex						
			Overall^†^		Males		Females		Male-to-female prevalence ratio^§^
			Prevalence	95% CI	Prevalence	95% CI	Prevalence	95% CI	
Arizona	**24,952**	**349**	14.0	(12.6–15.5)	21.1	(18.7–23.8)	6.6	(5.3–8.2)	3.2
Arkansas	**39,992**	**522**	13.1	(12.0–14.2)	20.5	(18.6–22.5)	5.4	(4.5–6.5)	3.8
Colorado	**41,128**	**572**	13.9	(12.8–15.1)	21.8	(19.9–23.9)	5.5	(4.6–6.7)	3.9
Georgia	**51,161**	**869**	17.0	(15.9–18.2)	27.9	(25.9–30.0)	5.7	(4.8–6.7)	4.9
Maryland	**9,955**	**199**	20.0	(17.4–23.0)	32.7	(28.1–38.2)	7.2	(5.2–10.0)	4.5
Minnesota	**9,767**	**234**	24.0	(21.1–27.2)	39.0	(33.8–44.9)	8.5	(6.3–11.6)	4.6
Missouri	**25,333**	**356**	14.1	(12.7–15.6)	22.2	(19.8–25.0)	5.6	(4.4–7.0)	4.0
New Jersey	**32,935**	**964**	29.3	(27.5–31.2)	45.5	(42.4–48.9)	12.3	(10.7–14.1)	3.7
North Carolina	**30,283**	**527**	17.4	(16.0–19.0)	28.0	(25.5–30.8)	6.5	(5.3–7.9)	4.3
Tennessee	**24,940**	**387**	15.5	(14.0–17.1)	25.3	(22.6–28.2)	5.4	(4.2–6.9)	4.7
Wisconsin	**35,037**	**494**	14.1	(12.9–15.4)	21.4	(19.4–23.7)	6.4	(5.3–7.7)	3.4
**All sites combined**	**325,483**	**5,473**	**16.8**	**(16.4**–**17.3)**	**26.6**	**(25.8**–**27.4)**	**6.6**	**(6.2**–**7.0)**	**4.0**

**Abbreviations:** ASD = autism spectrum disorder; CI = confidence interval.

* Per 1,000 children aged 8 years.

^†^ All children are included in the total regardless of race or ethnicity.

^§^ All sites identified significantly higher prevalence among males compared with females (p<0.01).

### Prevalence by Sex and Race/Ethnicity

When data from all 11 ADDM sites were combined, ASD prevalence was 26.6 per 1,000 boys and 6.6 per 1,000 girls (prevalence ratio: 4.0). ASD prevalence was significantly (p<0.01) higher among boys than among girls in all 11 ADDM sites (Table 2), with male-to-female prevalence ratios ranging from 3.2 (Arizona) to 4.9 (Georgia). Estimated ASD prevalence also varied by race and ethnicity (Table 3). When data from all sites were combined, the estimated prevalence among white children (17.2 per 1,000) was 7% greater than that among black children (16.0 per 1,000) and 22% greater than that among Hispanic children (14.0 per 1,000). In nine sites, the estimated prevalence of ASD was higher among white children than black children. The white-to-black ASD prevalence ratios were statistically significant in three sites (Arkansas, Missouri, and Wisconsin), and the white-to-Hispanic prevalence ratios were significant in seven sites (Arizona, Arkansas, Colorado, Georgia, Missouri, North Carolina, and Tennessee). In nine sites (Arizona, Arkansas, Colorado, Georgia, Maryland, Minnesota, Missouri, North Carolina, and Tennessee), the estimated prevalence of ASD was higher among black children than that among Hispanic children. The black-to-Hispanic prevalence ratio was significant in three of these nine sites (Arizona, Georgia, and North Carolina). In New Jersey, there was almost no difference in ASD prevalence estimates among white, black, and Hispanic children. Estimates for Asian/Pacific Islander children ranged from 7.9 per 1,000 (Colorado) to 19.2 per 1,000 (New Jersey) with notably wide CIs.

**TABLE 3:** Estimated prevalence* of autism spectrum disorder among children aged 8 years, by race/ethnicity — Autism and Developmental Disabilities Monitoring Network, 11 sites, United States, 2014

Site	Race/Ethnicity								Prevalence ratio		
	White		Black		Hispanic		Asian/Pacific Islander		White-to- Black	White-to- Hispanic	Black-to- Hispanic
	Prevalence	95% CI	Prevalence	95% CI	Prevalence	95% CI	Prevalence	95% CI			
Arizona	16.2	(14.1–18.6)	19.5	(13.3–28.6)	10.3	(8.5–12.5)	10.3	(5.5–19.1)	0.8	1.6^§^	1.9^§^
Arkansas	13.9	(12.6–15.5)	10.4	(8.3–12.9)	8.4	(6.2–11.3)	14.2	(8.1–25.1)	1.3^†^	1.7^§^	1.2
Colorado	15.0	(13.5–16.7)	11.4	(8.0–16.2)	10.6	(9.0–12.5)	7.9	(4.8–12.9)	1.3	1.4^§^	1.1
Georgia	17.9	(16.0–20.2)	17.1	(15.4–18.9)	12.6	(10.6–15.0)	11.9	(8.9–16.1)	1.1	1.4^§^	1.4^§^
Maryland	19.5	(16.0–23.8)	16.5	(12.7–21.4)	15.7	(9.1–27.0)	13.9	(7.5–25.8)	1.2	1.2	1.1
Minnesota	24.3	(19.8–29.8)	27.2	(21.7–34.2)	20.9	(14.7–29.7)	17.8	(12.3–25.7)	0.9	1.2	1.3
Missouri	14.1	(12.4–16.0)	10.8	(8.6–13.6)	4.9	(2.2–10.9)	10.7	(5.8–20.0)	1.3^†^	2.9^†^	2.2
New Jersey	30.2	(27.4–33.3)	26.8	(23.3–30.9)	29.3	(26.2–32.9)	19.2	(13.9–26.6)	1.1	1.0	0.9
North Carolina	18.6	(16.5–20.9)	16.1	(13.5–19.2)	11.9	(9.3–15.2)	19.1	(13.7–26.8) (6.7–23.3)	1.2	1.6^§^	1.4^†^
Tennessee	16.1	(14.3–18.2)	12.5	(9.7–16.0)	10.5	(7.6–14.7)	12.5		1.3	1.5^†^	1.2
Wisconsin	15.2	(13.6–17.0)	11.3	(8.9–14.2)	12.5	(10.0–15.6)	10.2	(6.1–16.9)	1.3^†^	1.2	0.9
**All sites combined**	**17.2**	**(16.5–17.8)**	**16.0**	**(15.1–16.9)**	**14.0**	**(13.1–14.9)**	**13.5**	**(11.8–15.4)**	**1.1^†^**	**1.2^§^**	**1.1^§^**

**Abbreviation:** CI = confidence interval.

* Per 1,000 children aged 8 years.

^†^ Pearson chi-square test of prevalence ratio significant at p<0.05.

^§^ Pearson chi-square test of prevalence ratio significant at p<0.01.

### Intellectual Ability

Data on intellectual ability were reported for nine sites (Arizona, Arkansas, Colorado, Georgia, Maryland, Minnesota, New Jersey, North Carolina, and Tennessee) having information available for at least 70% of children who met the ASD case definition (range: 70.8% [Tennessee] to 89.2% [North Carolina]). The median age of children’s most recent IQ tests, on which the following results are based, was 73 months (6 years, 1 month). Data from these nine sites yielded accompanying data on intellectual ability for 3,714 (80.3%) of 4,623 children with ASD. This proportion did not differ by sex or race/ethnicity in any of the nine sites or when combining data from all nine sites. Among these 3,714 children, 31% were classified in the range of ID (IQ ≤70), 25% were in the borderline range (IQ 71–85), and 44% had IQ >85. The proportion of children classified in the range of ID ranged from 26.7% in Arizona to 39.4% in Tennessee.

**FIGURE 1:**
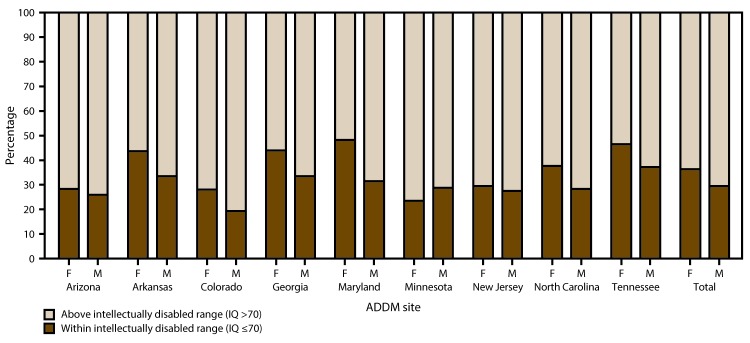
Most recent intelligence quotient score as of age 8 years among children with autism spectrum disorder for whom test data were available, by sex and site — Autism and Developmental Disabilities Monitoring Network, nine sites,* United States, 2014 **Abbreviations:** ADDM =Autism and Developmental Disabilities Monitoring Network; ASD= autism spectrum disorder; F = female; IQ = intelligence quotient; M = male. * Includes nine sites (Arizona, Arkansas, Colorado, Georgia, Maryland, Minnesota, New Jersey, North Carolina, and Tennessee) that had intellectual ability data available for ≥70% of children who met the ASD case definition (n = 3,714).

Among children identified with ASD, the distribution by intellectual ability varied by sex, with girls more likely than boys to have IQ ≤70, and boys more likely than girls to have IQ >85 (Figure 1). In these nine sites combined, 251 (36.3%) of 691 girls with ASD had IQ scores or examiners’ statements indicating ID compared with 891 (29.5%) of 3,023 males (odds ratio [OR] = 1.4; p<0.01), though among individual sites this proportion differed significantly in only one (Georgia, OR = 1.6; p<0.05). The proportion of children with ASD with borderline intellectual ability (IQ 71–85) did not differ by sex, whereas a significantly higher proportion of males (45%) compared with females (40%) had IQ >85 (i.e., average or above average intellectual ability) (OR = 1.2; p<0.05).

**FIGURE 2:**
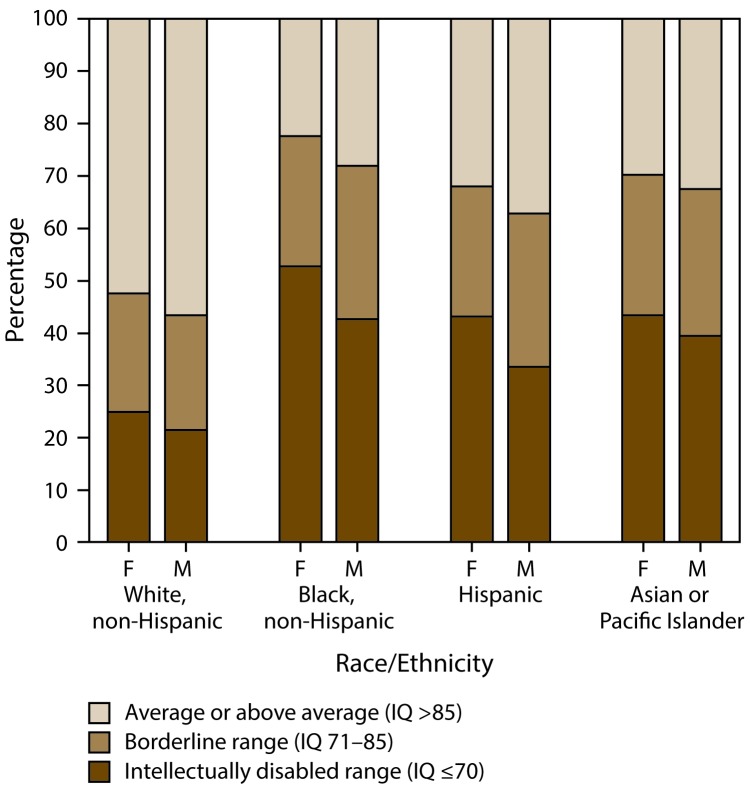
Most recent intelligence quotient score as of age 8 years among children with autism spectrum disorder for whom test data were available, by sex and race/ethnicity — Autism and Developmental Disabilities Monitoring Network, nine sites,* United States, 2014 **Abbreviations:** ASD = autism spectrum disorder; F = female; IQ = intelligence quotient; M = male. * Includes nine sites (Arizona, Arkansas, Colorado, Georgia, Maryland, Minnesota, New Jersey, North Carolina, and Tennessee) that had intellectual ability data available for ≥70 of children who met the ASD case definition (n = 3,714).

The distribution of intellectual ability also varied by race/ethnicity. Approximately 44% of black children with ASD were classified in the range of ID compared with 35% of Hispanic children and 22% of white children (Figure 2). The proportion of blacks and whites with ID differed significantly in all sites except Colorado, and when combining their data (OR = 2.9; p<0.01). The proportion of Hispanics and whites with ID differed significantly when combining data from all nine sites (OR = 1.9; p<0.01), and among individual sites it reached significance (p<0.05) in six of the nine sites, with the three exceptions being Arkansas (OR = 1.8; p = 0.10), North Carolina (OR = 1.8; p = 0.07), and Tennessee (OR = 2.1; p = 0.09). The proportion of children with borderline intellectual ability (IQ = 71–85) did not differ between black and Hispanic children, although a lower proportion of white children (22%) were classified in the range of borderline intellectual ability compared to black (28.4%; OR = 0.7; p<0.01) or Hispanic (28.7%; OR = 0.7; p<0.01) children. When combining data from these nine sites, the proportion of white children (56%) with IQ >85 was significantly higher than the proportion of black (27%, OR = 3.4; p<0.01) or Hispanic (36%, OR = 2.2; p<0.01) children with IQ>85.

### First Comprehensive Evaluation

Among children with ASD who were born in the same state as the ADDM site (n = 4,147 of 5,473 confirmed cases), 42% had a comprehensive evaluation on record by age 36 months (range: 30% [Arkansas] to 66% [North Carolina]) (Table 4). Approximately 39% of these 4,147 children did not have a comprehensive evaluation on record until after age 48 months; however, mention of developmental concerns by age 36 months was documented for 85% (range: 61% [Tennessee] to 94% [Arizona]).

**TABLE 4:** Number and percentage of children aged 8 years* identified with autism spectrum disorder who received a comprehensive evaluation by a qualified professional at age ≤36 months, 37–48 months, or >48 months, and those with a mention of general delay concern by age 36 months — Autism and Developmental Disabilities Monitoring Network, 11 sites, United States, 2014

Site	Earliest age when child received a comprehensive evaluation						Mention of general developmental delay	
	≤36 mos		37–48 mos		>48 mos		≤36 mos	
	No.	(%)	No.	(%)	No.	(%)	No.	(%)
Arizona	87	(34.1)	56	(22.0)	112	(43.9)	240	(94.1)
Arkansas	117	(30.5)	98	(25.6)	168	(43.9)	354	(92.4)
Colorado	200	(46.4)	66	(15.3)	165	(38.3)	383	(88.9)
Georgia	240	(37.6)	126	(19.7)	273	(42.7)	549	(85.9)
Maryland	96	(56.1)	19	(11.1)	56	(32.7)	158	(92.4)
Minnesota	57	(33.5)	36	(21.2)	77	(45.3)	124	(72.9)
Missouri	88	(32.1)	39	(14.2)	147	(53.6)	196	(71.5)
New Jersey	318	(40.5)	174	(22.2)	293	(37.3)	645	(82.2)
North Carolina	260	(66.2)	42	(10.7)	91	(23.2)	364	(92.6)
Tennessee	80	(34.0)	47	(20.0)	108	(46.0)	144	(61.3)
Wisconsin	194	(47.2)	87	(21.2)	130	(31.6)	368	(89.5)
**All sites combined**	**1,737**	**(41.9)**	**790**	**(19.0)**	**1,620**	**(39.1)**	**3,525**	**(85.0)**

* Includes children identified with autism spectrum disorder who were linked to an in-state birth certificate.

### Previously Documented ASD Classification

Of the 5,473 children meeting the ADDM ASD surveillance case definition, 4,379 (80%) had either eligibility for autism special education services or a DSM-IV-TR, DSM-5, or ICD-9 autism diagnosis documented in their records (range among 11 sites: 58% [Colorado] to 92% [Missouri]). Combining data from all 11 sites, 81% of boys had a previous ASD classification on record, compared with 75% of girls (OR = 1.4; p<0.01). When stratified by race/ethnicity, 80% of white children had a previously documented ASD classification, compared with nearly 83% of black children (OR = 0.9; p = 0.09) and 76% of Hispanic children (OR = 1.3; p<0.01); a significant difference was also found when comparing the proportion of black children with a previous ASD classification to that among Hispanic children (OR = 1.5; p<0.01).

The median age of earliest known ASD diagnosis documented in children’s records (Table 5) varied by diagnostic subtype (autistic disorder: 46 months; ASD/PDD: 56 months; Asperger disorder: 67 months). Within these subtypes, the median age of earliest known diagnosis did not differ by sex, nor did any difference exist in the proportion of boys and girls who initially received a diagnosis of autistic disorder (48%), ASD/PDD (46%), or Asperger disorder (6%). The median age of earliest known diagnosis and distribution of subtypes did vary by site. The median age of earliest known ASD diagnosis for all subtypes combined was 52 months, ranging from 40 months in North Carolina to 59 months in Arkansas.

**TABLE 5:** Median age (in months) of earliest known autism spectrum disorder diagnosis and number and proportion within each diagnostic subtype — Autism and Developmental Disabilities Monitoring Network, 11 sites, United States, 2014

Site	Autistic disorder			ASD/PDD			Asperger disorder			Any specified ASD diagnosis		
	Median age	No.	(%)	Median age	No.	(%)	Median age	No.	(%)	Median age	No.	(%)
Arizona	55	186	(76.2)	61	50	(20.5)	74	8	(3.3)	56	244	(69.9)
Arkansas	55	269	(63.0)	63	129	(30.2)	75	29	(6.8)	59	427	(81.8)
Colorado	40	192	(61.7)	65	104	(33.4)	61	15	(4.8)	51	311	(54.4)
Georgia	46	288	(48.1)	56	261	(43.6)	65	50	(8.3)	53	599	(68.9)
Maryland	43	52	(32.3)	61	104	(64.6)	65	5	(3.1)	52	161	(80.9)
Minnesota	51	50	(45.9)	65	54	(49.5)	62	5	(4.6)	56	109	(46.6)
Missouri	54	81	(26.7)	55	197	(65.0)	65	25	(8.3)	56	303	(85.1)
New Jersey	42	227	(32.7)	51	428	(61.6)	66	40	(5.8)	48	695	(72.1)
North Carolina	32	165	(52.5)	49	130	(41.4)	67	19	(6.1)	40	314	(59.6)
Tennessee	51	157	(57.1)	63	100	(36.4)	60	18	(6.5)	56	275	(71.1)
Wisconsin	46	143	(40.2)	55	189	(53.1)	67	24	(6.7)	51	356	(72.1)
**All sites combined**	**46**	**1,810**	**(47.7)**	**56**	**1,746**	**(46.0)**	**67**	**238**	**(6.3)**	**52**	**3,794**	**(69.3)**

**Abbreviations:** ASD = autism spectrum disorder; PDD = pervasive developmental disorder–not otherwise specified.

### Special Education Eligibility

Sites with access to education records collected information on the most recent eligibility categories under which children received special education services (Table 6). Among children with ASD who were receiving special education services in public schools during 2014, the proportion of children with a primary eligibility category of autism ranged from approximately 37% in Wisconsin to 80% in Tennessee. Most other sites noted approximately 60% to 75% of children with ASD having autism listed as their most recent primary special education eligibility category, the exceptions being Colorado (44%) and New Jersey (48%). Other common special education eligibilities included health or physical disability, speech and language impairment, specific learning disability, and a general developmental delay category that is used until age 9 years in many U.S. states. All ADDM sites reported <10% of children with ASD receiving special education services under a primary eligibility category of ID.

**TABLE 6:** Number and percentage of children aged 8 years identified with autism spectrum disorder with available special education records, by primary special education eligibility category* — Autism and Developmental Disabilities Monitoring Network, 10 sites, United States, 2014

Characteristic	Arizona	Arkansas	Colorado	Georgia	Maryland	Minnesota	New Jersey	North Carolina	Tennessee	Wisconsin
Total no. of ASD cases	349	522	572	869	199	234	964	527	387	494
Total no. (%) of ASD cases with special education records	308 (88.3)	327^† ^(—^§^)	139^† ^(—^§^)	708 (81.5)	149 (74.9)	188 (80.3)	822 (85.3)	420 (79.7)	218^† ^(—^§^)	156^† ^(—^§^)
**Primary exceptionality (%)**										
Autism	64.9	65.4	43.9	58.9	67.1	67.0	48.4	75.0	79.8	36.5
Emotional disturbance	2.9	0.9	7.2	2.0	2.7	3.7	1.6	2.6	0.5	5.8
Specific learning disability	6.8	3.7	13.7	4.0	12.8	1.1	8.2	2.9	0.9	2.6
Speech or language impairment	5.5	8.9	10.8	1.0	3.4	2.7	13.7	2.4	3.2	20.5
Hearing or visual impairment	0	0.3	0	0.1	0	1.1	0.6	0.5	0	0.6
Health, physical or other disability	6.8	13.5	14.4	3.5	8.1	15.4	18.5	11.2	3.2	14.7
Multiple disabilities	0.3	3.4	5.0	0	4.0	1.6	6.7	1.7	0	0
Intellectual disability	3.2	4.0	4.3	2.0	2.0	6.9	1.7	2.4	2.8	0.6
Developmental delay/Preschool	9.4	0	0.7	28.5	0	0.5	0.6	1.4	9.6	18.6

**Abbreviation:** ASD = autism spectrum disorder.

* Some state-specific categories were recoded or combined to match current U.S. Department of Education categories.

^†^ Excludes children residing in school districts where educational records were not reviewed (proportion of surveillance population: 31% Arkansas, 67% Colorado, 12% Tennessee, 74% Wisconsin).

^§^ Proportion not reported because numerator is not comparable to other sites (excludes children residing in school districts where educational records were not reviewed).

### Sensitivity Analyses of Missing Records and Expanded ICD-9 Codes

A stratified analysis of records that could not be located for review was completed to assess the degree to which missing data might have potentially reduced prevalence estimates as reported by individual ADDM sites. Had all children’s records identified in Phase 1 been located and reviewed, prevalence estimates would potentially have been <1% higher in four sites (Arizona, Georgia, Minnesota, and Wisconsin), between 1% to 5% higher in four sites (Colorado, Missouri, New Jersey, and North Carolina), approximately 8% higher in Maryland, and nearly 20% higher in Arkansas and Tennessee, where investigators were able to access education records throughout most, but not all, of the surveillance area and received data from their state Department of Education to evaluate the potential impact on reported ASD prevalence estimates attributed to missing records.

The impact on prevalence estimates of reviewing records based on an expanded list of ICD-9 codes varied from site to site. Colorado, Georgia, and Missouri were the only three sites that identified more than 1% of ASD surveillance cases partially or solely on the basis of the expanded code list. In Missouri, less than 2% of children identified with ASD had some of their records located on the basis of the expanded code list, and none were identified exclusively from these codes. In Colorado, approximately 2% of ASD surveillance cases had some abstracted records identified on the basis of the expanded code list, and 4% had records found exclusively from the expanded codes. In Georgia, where ICD-9 codes were requested for surveillance of five distinct conditions (autism, cerebral palsy, ID, hearing loss, and vision impairment), approximately 10% of children identified with ASD had some of their records located on the basis of the expanded code list, and less than 1% were identified exclusively from these codes.

### Comparison of Case Counts from DSM-IV-TR and DSM-5 Case Definitions

The DSM-5 analysis was completed for part of the overall ADDM 2014 surveillance area (Table 7), representing a total population of 263,775 children aged 8 years. This was 81% of the population on which DSM-IV-TR prevalence estimates were reported. Within this population, 4,920 children were confirmed to meet the ADDM Network ASD case definition for either DSM-IV-TR or DSM-5. Of these children, 4,236 (86%) met both case definitions, 422 (9%) met only the DSM-IV-TR criteria, and 262 (5%) met only the DSM-5 criteria (Table 8). This yielded a DSM-IV-TR:DSM-5 prevalence ratio of 1.04 in this population, indicating that ASD prevalence was approximately 4% higher based on the historical DSM-IV-TR case definition compared with the new DSM-5 case definition. Among 4,498 children who met DSM-5 case criteria, 3,817 (85%) met the DSM-5 behavioral criteria (Box 2), whereas 681 (15%) qualified on the basis of an established ASD diagnosis but did not have sufficient DSM-5 behavioral criteria documented in comprehensive evaluations. In six of the 11 ADDM sites, DSM-5 case counts were within approximately 5% of DSM-IV-TR counts (range: 5% lower [Tennessee] to 5% higher [Arkansas]), whereas DSM-5 case counts were more than 5% lower than DSM-IV-TR counts in Minnesota and North Carolina (6%), New Jersey (10%), and Colorado (14%). Kappa statistics indicated strong agreement between DSM-IV-TR and DSM-5 case status among children abstracted in Phase 1 of the study who were reviewed in Phase 2 for both DSM-IV-TR and DSM-5 (kappa for all sites combined: 0.85, range: 0.72 [Tennessee] to 0.93 [North Carolina]).

**TABLE 7:** Number* and percentage of children aged 8 years, by race/ethnicity and site in the DSM-5 Surveillance Area — Autism and Developmental Disabilities Monitoring Network, 11 sites, United States, 2014

Site	Site institution	Surveillance area	Total	White, non-Hispanic		Black, non-Hispanic		Hispanic		Asian or Pacific Islander, non-Hispanic		American Indian or Alaska Native, non-Hispanic	
			No.	No.	(%)	No.	(%)	No.	(%)	No.	(%)	No.	(%)
Arizona	University of Arizona	Part of 1 county in metropolitan Phoenix^†^	**9,478**	5,340	(56.3)	321	(3.4)	3,244	(34.2)	296	(3.1)	277	(2.9)
Arkansas	University of Arkansas for Medical Sciences	All 75 counties in Arkansas	**39,992**	26,103	(65.3)	7,705	(19.3)	5,012	(12.5)	843	(2.1)	329	(0.8)
Colorado	Colorado Department of Public Health and Environment	1 county in metropolitan Denver	**8,022**	2,603	(32.4)	1,018	(12.7)	4,019	(50.1)	322	(4.0)	60	(0.7)
Georgia	CDC	5 counties including metropolitan Atlanta	**51,161**	15,495	(30.3)	22,042	(43.1)	9,913	(19.4)	3,599	(7.0)	112	(0.2)
Maryland	Johns Hopkins University	1 county in metropolitan Baltimore	**9,955**	4,977	(50.0)	3,399	(34.1)	829	(8.3)	719	(7.2)	31	(0.3)
Minnesota	University of Minnesota	Parts of 2 counties including Minneapolis–St. Paul^†^	**9,767**	3,793	(38.8)	2,719	(27.8)	1,486	(15.2)	1,576	(16.1)	193	(2.0)
Missouri	Washington University	1 county in metropolitan St. Louis	**12,205**	7,186	(58.9)	3,793	(31.1)	561	(4.6)	626	(5.1)	39	(0.3)
New Jersey	Rutgers University	4 counties including metropolitan Newark	**32,935**	13,593	(41.3)	7,166	(21.8)	10,226	(31.0)	1,874	(5.7)	76	(0.2)
North Carolina	University of North Carolina–Chapel Hill	6 counties in central North Carolina	**30,283**	15,241	(50.3)	7,701	(25.4)	5,463	(18.0)	1,778	(5.9)	100	(0.3)
Tennessee	Vanderbilt University Medical Center	11 counties in middle Tennessee	**24,940**	15,867	(63.6)	4,896	(19.6)	3,324	(13.3)	799	(3.2)	54	(0.2)
Wisconsin	University of Wisconsin–Madison	10 counties in southeastern Wisconsin	**35,037**	20,732	(59.2)	6,486	(18.5)	6,181	(17.6)	1,471	(4.2)	167	(0.5)
**All sites combined**			**263,775**	**130,930**	**(49.6)**	**67,246**	**(25.5)**	**50,258**	**(19.1)**	**13,903**	**(5.3)**	**1,438**	**(0.5)**

**Abbreviation:** DSM-5 = *Diagnostic and Statistical Manual of Mental Disorders, Fifth Edition*.

* Total numbers of children aged 8 years in each surveillance area were obtained from CDC’s National Center for Health Statistics Vintage 2016 Bridged-Race Population Estimates for July 1, 2014.

^†^ Denominator excludes school districts that were not included in the surveillance area, calculated from National Center for Education Statistics enrollment counts of third graders during the 2014–2015 school year.

**TABLE 8:** Number and percentage of children meeting DSM-IV-TR and/or DSM-5 surveillance case definition — Autism and Developmental Disabilities Monitoring Network, 11 sites, United States, 2014

Site	Met DSM-IV-TR or DSM-5	Met both DSM-IV-TR and DSM-5		Met DSM-IV-TR only		Met DSM-5 only		DSM-IV-TR vs. DSM-5	
	No.	No.	(%)	No.	(%)	No.	(%)	Ratio	Kappa
Arizona	179	143	(79.9)	17	(9.5)	19	(10.6)	0.99	0.83
Arkansas	560	514	(91.8)	8	(1.4)	38	(6.8)	0.95	0.92
Colorado	116	92	(79.3)	19	(16.4)	5	(4.3)	1.14	0.79
Georgia	937	790	(84.3)	79	(8.4)	68	(7.3)	1.01	0.83
Maryland	207	187	(90.3)	12	(5.8)	8	(3.9)	1.02	0.89
Minnesota	254	200	(78.7)	34	(13.4)	20	(7.9)	1.06	0.79
Missouri	209	179	(85.6)	12	(5.7)	18	(8.6)	0.97	0.74
New Jersey	995	842	(84.6)	122	(12.3)	31	(3.1)	1.10	0.85
North Carolina	532	493	(92.7)	34	(6.4)	5	(0.9)	1.06	0.93
Tennessee	408	348	(85.3)	39	(9.6)	21	(5.1)	1.05	0.72
Wisconsin	523	448	(85.7)	46	(8.8)	29	(5.5)	1.04	0.83
**All sites combined**	**4,920**	**4,236**	**(86.1)**	**422**	**(8.6)**	**262**	**(5.3)**	**1.04**	**0.85**

**Abbreviations:** DSM-5 = *Diagnostic and Statistical Manual of Mental Disorders, Fifth Edition*; DSM-IV-TR = *Diagnostic and Statistical Manual of Mental Disorders, Fourth Edition, Text Revision*.

Stratified analysis of DSM-IV-TR:DSM-5 ratios were very similar compared with the overall sample (Table 9). DSM-5 estimates were approximately 3% lower than DSM-IV-TR counts for males, and approximately 6% lower for females (kappa = 0.85 for both). Case counts were approximately 3% lower among white and black children on DSM-5 compared with DSM-IV-TR, 5% lower among Asian children, and 8% lower among Hispanic children. Children who received a comprehensive evaluation by age 36 months were 7% less likely to meet DSM-5 than DSM-IV-TR, whereas those evaluated by age 4 years were 6% less likely to meet DSM-5, and those initially evaluated after age 4 years were just as likely to meet DSM-5 as DSM-IV-TR. Children with documentation of eligibility for autism special education services, and those with a documented diagnosis of ASD by age 3 years, were 2% more likely to meet DSM-5 than DSM-IV-TR. Slightly over 3% of children whose earliest ASD diagnosis was autistic disorder met DSM-5 criteria but not DSM-IV-TR, compared with slightly under 3% of those whose earliest diagnosis was PDD-NOS/ASD-NOS and 5% of those whose earliest diagnosis was Asperger disorder. Children with no previous ASD classification (diagnosis or eligibility) were 47% less likely to meet DSM-5 than DSM-IV-TR. Combining data from all 11 sites, children with IQ scores in the range of ID were 3% less likely to meet DSM-5 criteria compared with DSM-IV-TR (kappa = 0.89), those with IQ scores in the borderline range were 6% less likely to meet DSM-5 than DSM-IV-TR (kappa = 0.88), and children with average or above average intellectual ability were 4% less likely to meet DSM-5 criteria compared with DSM-IV-TR (kappa = 0.86).

**TABLE 9:** Characteristics of children meeting DSM-IV-TR and/or DSM-5 surveillance case definition — Autism and Developmental Disabilities Monitoring Network, 11 sites, United States, 2014

Characteristic	Met DSM-IV-TR or DSM-5	Met both DSM-IV-TR and DSM-5		Met DSM-IV-TR only		Met DSM-5 only		DSM-IV-TR vs. DSM-5	
	No.	No.	(%)	No.	(%)	No.	(%)	Ratio	Kappa
**Met ASD case definition under DSM-IV-TR and/or DSM-5**	4,920	4,236	(86.1)	422	(8.6)	262	(5.3)	1.04	0.85
Male	3,978	3,452	(86.8)	316	(7.9)	210	(5.3)	1.03	0.85
Female	942	784	(83.2)	106	(11.3)	52	(5.5)	1.06	0.85
White, non-Hispanic	2,486	2,159	(86.8)	193	(7.8)	134	(5.4)	1.03	0.85
Black, non-Hispanic	1,184	994	(84.0)	109	(9.2)	81	(6.8)	1.03	0.84
Hispanic, regardless of race	817	695	(85.1)	91	(11.1)	31	(3.8)	1.08	0.86
Asian/Pacific Islander, non-Hispanic	207	188	(90.8)	14	(6.8)	5	(2.4)	1.05	0.88
≤36 months	1,509	1,372	(90.9)	115	(7.6)	22	(1.5)	1.07	0.89
37–48 months	723	640	(88.5)	61	(8.4)	22	(3.0)	1.06	0.86
>48 months	1,503	1,195	(79.5)	154	(10.2)	154	(10.2)	1.00	0.81
Autism special education eligibility^†^	2,270	2,156	(95.0)	35	(1.5)	79	(3.5)	0.98	0.57
**ASD diagnostic statement^§^**									
Earliest ASD diagnosis ≤36 months	951	936	(98.4)	0	(0)	15	(1.6)	0.98	0.71
Earliest ASD diagnosis autistic disorder	1,577	1,526	(96.8)	0	(0)	51	(3.2)	0.97	0.50
Earliest ASD diagnosis PDD-NOS/ASD-NOS	1,564	1,525	(97.5)	0	(0)	39	(2.5)	0.98	0.72
Earliest ASD diagnosis Asperger disorder	221	210	(95.0)	0	(0)	11	(5.0)	0.95	0.72
No previous ASD diagnosis or eligibility on record	950	484	(50.9)	369	(38.8)	97	(10.2)	1.47	0.62
Intellectual disability (IQ ≤70)	1,191	1,089	(91.4)	67	(5.6)	35	(2.9)	1.03	0.89
Borderline range (IQ 71–85)	881	778	(88.3)	74	(8.4)	29	(3.3)	1.06	0.88
Average or above average (IQ >85)	1,620	1,391	(85.9)	143	(8.8)	86	(5.3)	1.04	0.86

**Abbreviations:** ASD = autism spectrum disorder; DSM-5 = *Diagnostic and Statistical Manual of Mental Disorders, Fifth Edition*; DSM-IV-TR = *Diagnostic and Statistical Manual of Mental Disorders, Fourth Edition, Text Revision*; PDD-NOS = pervasive developmental disorder–not otherwise specified.

* Includes children identified with ASD who were linked to an in-state birth certificate.

^†^ Includes children with autism as the Primary Exceptionality (Table 6) as well as children documented to meet eligibility criteria for autism special education services.

^§^ An ASD diagnosis documented in abstracted comprehensive evaluations, including DSM-IV-TR diagnosis of autistic disorder, PDD-NOS or Asperger disorder qualifies a child as meeting the DSM-5 surveillance case definition for ASD.

^¶^ Includes data from all 11 sites, including those with IQ data available for <70% of confirmed cases.

## Discussion

### Changes in Estimated Prevalence

The overall ASD prevalence estimate of 16.8 per 1,000 children aged 8 years in 2014 is higher than previously reported estimates from the ADDM Network. An ASD case definition based on DSM-IV-TR criteria was used during the entire period of ADDM surveillance during 2000–2014, as were comparable study operations and procedures, although the geographic areas under surveillance have varied over time. During this period, ADDM ASD prevalence estimates increased from 6.7 to 16.8 per 1,000 children aged 8 years, an increase of approximately 150%.

Among the six ADDM sites completing both the 2012 and 2014 studies for the same geographic area, all six showed higher ASD prevalence estimates for 2012 compared to 2014, with a nearly 10% higher prevalence in Georgia (p = 0.06) and Maryland (p = 0.35), 19% in New Jersey (p<0.01), 22% in Missouri (p = 0.01), 29% in Colorado (p<0.01), and 31% in Wisconsin (p<0.01). When combining data from these six sites, ASD prevalence estimates for 2014 were 20% higher for 2014 compared to 2012 (p<0.01). The ASD prevalence estimate from New Jersey continues to be one of the highest reported by a population-based surveillance system. The two sites with the greatest relative difference in prevalence are noteworthy in that both gained access to children’s education records in additional geographic areas for 2014. Colorado was granted access to review children’s education records in one additional county for the 2014 surveillance year (representing nearly 20% of the population aged 8 years within the overall Colorado surveillance area), and Wisconsin was granted access to review education records for more than a quarter of its surveillance population, and 2014 marked the first time Wisconsin has included education data sources. Comparisons with earlier ADDM Network surveillance results should be interpreted cautiously because of changing composition of sites and geographic coverage over time. For example, three ADDM Network sites completing both the 2012 and 2014 surveillance years (Arizona, Arkansas, and North Carolina) covered a different geographic area each year, and two new sites (Minnesota and Tennessee) were awarded funding to monitor ASD in collaboration with the ADDM Network.

Certain characteristics of children with ASD were similar in 2014 compared with earlier surveillance years. The median age of earliest known ASD diagnosis remained close to 53 months in previous surveillance years and was 52 months in 2014. The proportion of children who received a comprehensive developmental evaluation by age 3 years was unchanged: 42% in 2014 and 43% during 2006–2012. There were a number of differences in the characteristics of the population of children with ASD in 2014. The male:female prevalence ratio decreased from 4.5:1 during 2002–2012 to 4:1 in 2014, driven by a greater relative increase in ASD prevalence among girls than among boys since 2012. Also, the decrease in the ratios of white:black and white:Hispanic children with ASD continued a trend observed since 2002. Among sites covering a population of at least 20,000 children aged 8 years, New Jersey reported no significant race- or ethnicity-based difference in ASD prevalence, suggesting more complete ascertainment among all children regardless of race/ethnicity. Historically, ASD prevalence estimates from combined ADDM sites have been approximately 20%–30% higher among white children as compared with black children. For surveillance year 2014, the difference was only 7%, the lowest difference ever observed for the ADDM Network. Likewise, prevalence among white children was almost 70% higher than that among Hispanic children in 2002 and 2006, and approximately 50% higher in 2008, 2010, and 2012, whereas for 2014 the difference was only 22%. Data from a previously reported comparison of ADDM Network ASD prevalence estimates from 2002, 2006, and 2008 (*9*) suggested greater increases in ASD prevalence among black and Hispanic children compared with those among white children. Reductions in disparities in ASD prevalence for black and Hispanic children might be attributable, in part, to more effective outreach directed to minority communities. Finally, the proportion of children with ASD and lower intellectual ability was similar in 2012 and 2014 at approximately 30% of males and 35% of females. These proportions were markedly lower than those reported in previous surveillance years.

### Variation in Prevalence Among ADDM Sites

Findings from the 2014 surveillance year indicate that prevalence estimates still vary widely among ADDM Network sites, with the highest prevalence observed in New Jersey. Although five of the 11 ADDM sites conducting the 2014 surveillance year reported prevalence estimates within a very close range (from 13.1 to 14.1 per 1,000 children), New Jersey’s prevalence estimate of 29.3 per 1,000 children was significantly greater than that from any other site, and four sites (Georgia, Maryland, Minnesota, and North Carolina) reported prevalence estimates that were significantly greater than those from any of the five sites in the 13.1–14.1 per 1,000 range. Two of the sites with prevalence estimates of 20.0 per 1,000 or higher (Maryland and Minnesota) conducted surveillance among a total population of <10,000 children aged 8 years. Concentrating surveillance efforts in smaller geographic areas, especially those in close proximity to diagnostic centers and those covering school districts with advanced staff training and programs to support children with ASD, might yield higher prevalence estimates compared with those from sites covering populations of more than 20,000 children aged 8 years. Of the six sites with prevalence estimates below the 16.8 per 1,000 estimate for all sites combined, five did not have full access to education data sources (Arkansas, Colorado, Missouri, Tennessee, and Wisconsin), whereas only one of the six sites will full access to education data sources had a prevalence estimate below 16.8 per 1,000 (Arizona). Such differences cannot be attributed solely to source access, as other factors (e.g., demographic differences and service availability) also might have influenced these findings. In addition to variation among sites in reported ASD prevalence, wide variation among sites is noted in the characteristics of children identified with ASD, including the proportion of children who received a comprehensive developmental evaluation by age 3 years, the median age of earliest known ASD diagnosis, and the distribution by intellectual ability. Some of this variation might be attributable to regional differences in diagnostic practices and other documentation of autism symptoms, although previous reports based on ADDM data have linked much of the variation to other extrinsic factors, such as regional and socioeconomic disparities in access to services (*13*,*14*).

### Case Definitions

Results from application of the DSM-IV-TR and DSM-5 case definitions were similar, overall and when stratified by sex, race/ethnicity, DSM-IV-TR diagnostic subtype, or level of intellectual ability. Overall, ASD prevalence estimates based on the new DSM-5 case definition were very similar in magnitude but slightly lower than those based on the historical DSM-IV-TR case definition. Three of the 11 ADDM sites had slightly higher case counts using the DSM-5 framework compared with the DSM-IV-TR. Colorado, where the DSM-IV-TR:DSM-5 ratio was highest compared with all other sites, was also the site with the lowest proportion of DSM-IV-TR cases having a previous ASD classification. This suggests that the diagnostic component of the DSM-5 case definition, whereby children with a documented diagnosis of ASD might qualify as DSM-5 cases regardless of social interaction/communication and restricted/repetitive behavioral criteria, might have influenced DSM-5 results to a lesser degree in that site, as a smaller proportion of DSM-IV-TR cases would meet DSM-5 case criteria based solely on the presence of a documented ASD diagnosis. This element of the DSM-5 case definition might carry less weight moving forward, as fewer children aged 8 years in health and education settings will have had ASD diagnosed under the DSM-IV-TR criteria. It is also possible that persons who conduct developmental evaluations of children in health and education settings will increasingly describe behavioral characteristics using language more consistent with DSM-5 terminology, yielding more ASD cases based on the behavioral component of ADDM’s DSM-5 case definition. Prevalence estimates based on the DSM-5 case definition that incorporates an existing ASD diagnosis reflect the actual patterns of diagnosis and services for children in 2014, because children diagnosed under DSM-IV-TR did not lose their diagnosis when the updated DSM-5 criteria were published and because professionals might diagnose children with ASD without necessarily recording every behavior supporting that diagnosis. In the future, prevalence estimates will align more closely with the specific DSM-5 behavioral criteria, and might exclude some persons who would have met DSM-IV-TR criteria for autistic disorder, PDD-NOS, or Asperger disorder, while at the same time including persons who do not meet those criteria but who do meet the specific DSM-5 behavioral criteria.

### Comparison of Autism Prevalence Estimates

The ADDM Network is the only ASD surveillance system in the United States providing robust prevalence estimates for specific areas of the country, including those for subgroups defined by sex and race/ethnicity, providing information about geographical variation that can be used to evaluate policies and diagnostic practices that might affect ASD prevalence. It is also the only comprehensive surveillance system to incorporate ASD diagnostic criteria into the case definition rather than relying entirely on parent or caregiver report of a previous ASD diagnosis, providing a unique contribution to the knowledge of ASD epidemiology and the impact of changes in diagnostic criteria. Two surveys of children’s health, The National Health Interview Survey (NHIS) and the National Survey of Children’s Health (NSCH), report estimates of ASD prevalence based on caregiver report of being told by a doctor or other health care provider that their child has ASD, and, for the NSCH, if their child was also reported to currently have ASD. The most recent publication from NHIS indicated that 27.6 per 1,000 children aged 3–17 years had ASD in 2016, which did not differ significantly from estimates for 2015 or 2014 (24.1 and 22.4, respectively) (*28*). An estimate of 20.0 per 1,000 children aged 6–17 years was reported from the 2011–2012 NSCH (*29*). The study samples for both surveys are substantially smaller than the ADDM Network; however, they were intended to be nationally representative, whereas the ADDM Network surveillance areas were selected through a competitive process and, although large and diverse, were not intended to be nationally representative. Geographic differences in ASD prevalence have been observed in both the ADDM Network and national surveys, as have differences in ASD prevalence by age (*6*–*11*,*28*,*29*).

All three prevalence estimation systems (NHIS, NSCH, and ADDM) are subject to regional and policy-driven differences in the availability and utilization of evaluation and diagnostic services for children with developmental concerns. Phone surveys are likely more sensitive in identifying children who received a preliminary or confirmed diagnosis of ASD but are not receiving services (i.e., special education services). The ADDM Network method based on analysis of information contained in existing health and education records enables the collection of detailed, case-specific information reflecting children’s behavioral, developmental and functional characteristics, which are not available from the national phone surveys. This detailed case level information might provide insight into temporal changes in the expression of ASD phenotypes, and offers the ability to account for differences based on changing diagnostic criteria.

## Limitations

The findings in this report are subject to at least three limitations. First, ADDM Network sites were not selected to represent the United States as a whole, nor were the geographic areas within each ADDM site selected to represent that state as a whole (with the exception of Arkansas, where ASD is monitored statewide). Although a combined estimate is reported for the Network as a whole to inform stakeholders and interpret the findings from individual surveillance years in a more general context, data reported by the ADDM Network should not be interpreted to represent a national estimate of the number and characteristics of children with ASD. Rather, it is more prudent to examine the wide variation among sites, between specific groups within sites, and across time in the number and characteristics of children identified with ASD, and to use these findings to inform public health strategies aimed at removing barriers to identification and treatment, and eliminating disparities among socioeconomic and racial/ethnic groups. Data from individual sites provide even greater utility for developing local policies in those states.

Second, it is important to acknowledge limitations of information available in children’s health and education records when considering data on the characteristics of children with ASD. Age of earliest known ASD diagnosis was obtained from descriptions in children’s developmental evaluations that were available in the health and education facilities where ADDM staff had access to review records. Some children might have had earlier diagnoses that were not recorded in these records. Likewise, some descriptions of historical diagnoses (i.e., those not made by the evaluating examiner) could be subject to recall error by a parent or provider who described the historical diagnosis to that examiner. Another characteristic featured prominently in this report, intellectual ability, is subject to measurement limitations. IQ test results should be interpreted cautiously because of myriad factors that impact performance on these tests, particularly language and attention deficits that are common among children with ASD, especially when testing was conducted before age 6 years. Because children were not examined directly nor systematically by ADDM staff as part of this study, descriptions of their characteristics should not be interpreted to serve as the basis for policy changes, individual treatments, or interventions.

Third, because comparisons with the results from earlier ADDM surveillance years were not restricted to a common geographic area, inferences about the changing number and characteristics of children with ASD over time should be made with caution. Findings for each unique ADDM birth cohort are very informative, and although study methods and geographic areas of coverage have remained generally consistent over time, temporal comparisons are subject to multiple sources of bias and should not be misinterpreted as representing precise measures that control for all sources of bias. Additional limitations to the records-based surveillance methodology have been described extensively in previous ADDM and MADDSP reports (*3*,*6*–*11*).

## Future Surveillance Directions

Data collection for the 2016 surveillance year began in early 2017 and will continue through mid-2019. Beginning with surveillance year 2016, the DSM-5 case definition for ASD will serve as the basis for prevalence estimates. The DSM-IV-TR case definition will be applied in a limited geographic area to offer additional data for comparison, although the DSM-IV-TR case definition will eventually be phased out.

CDC’s “Learn the Signs. Act Early” (LTSAE) campaign, launched in October 2004, aims to change perceptions among parents, health care professionals, and early educators regarding the importance of early identification and treatment of autism and other developmental disorders (*30*). In 2007, the American Academy of Pediatrics (AAP) recommended developmental screening specifically focused on social development and ASD at age 18 and 24 months (*31*). Both efforts are in accordance with the *Healthy People 2020* (HP2020) goal that children with ASD be evaluated by age 36 months and begin receiving community-based support and services by age 48 months (*12*). It is concerning that progress has not been made toward the HP2020 goal of increasing the percentage of children with ASD who receive a first evaluation by age 36 months to 47%; however, the cohort of children monitored under the ADDM 2014 surveillance year (i.e., children born in 2006) represents the first ADDM 8-year-old cohort impacted by the LTSAE campaign and the 2007 AAP recommendations. The effect of these programs in lowering age at evaluation might become more apparent when subsequent birth cohorts are monitored. Further exploration of ADDM data, including those collected on cohorts of children aged 4 years (*32*), might inform how policy initiatives, such as screening recommendations and other social determinants of health, impact the prevalence of ASD and characteristics of children with ASD, including the age at which most children receive an ASD diagnosis.

## Conclusion

The latest findings from the ADDM Network provide evidence that the prevalence of ASD is higher than previously reported ADDM estimates and continues to vary among certain racial/ethnic groups and communities. The overall ASD prevalence estimate of 16.8 per 1,000 children aged 8 years in 2014 is higher than previous estimates from the ADDM Network. With prevalence of ASD reaching nearly 3% in some communities and representing an increase of 150% since 2000, ASD is an urgent public health concern that could benefit from enhanced strategies to help identify ASD earlier; to determine possible risk factors; and to address the growing behavioral, educational, residential and occupational needs of this population.

Implementation of the new DSM-5 case definition had little effect on the overall number of children identified with ASD for the ADDM 2014 surveillance year. This might be a result of including documented ASD diagnoses in the DSM-5 surveillance case definition. Over time, the estimate might be influenced (downward) by a diminishing number of persons who meet the DSM-5 diagnostic criteria for ASD based solely on a previous DSM-IV-TR diagnosis, such as autistic disorder, PDD-NOS or Asperger disorder, and influenced (upward) by professionals aligning their clinical descriptions with the DSM-5 criteria. Although the prevalence of ASD and characteristics of children identified by each case definition were similar in 2014, the diagnostic features defined under DSM-IV-TR and DSM-5 appear to be quite different. The ADDM Network will continue to evaluate these similarities and differences in much greater depth, and will examine at least one more cohort of children aged 8 years to expand this comparison. Over time, the ADDM Network will be well positioned to evaluate the effects of changing ASD diagnostic parameters on prevalence.
