# *Erratum*: Vol. 67, No. SS-6

**DOI:** 10.15585/mmwr.mm6719a8

**Published:** 2018-05-18

In the report “Prevalence of Autism Spectrum Disorder Among Children Aged 8 Years — Autism and Developmental Disabilities Monitoring Network, 11 Sites, United States, 2014,” on page 12, the first sentence of the second paragraph of the Discussion section should have read “Among the six ADDM sites completing both the 2012 and 2014 studies for the same geographic area, all six showed higher ASD prevalence estimates for **2014 compared to 2012**, with a nearly 10% higher prevalence in Georgia (p = 0.06) and Maryland (p = 0.35), 19% in New Jersey (p<0.01), 22% in Missouri (p = 0.01), 29% in Colorado (p<0.01), and 31% in Wisconsin (p<0.01).” 

On page 13, the second sentence under the heading “Variation in Prevalence Among ADDM Sites” should have read “Although five of the 11 ADDM sites conducting the 2014 surveillance year reported prevalence estimates within a very close range (from 13.1 to 14.1 per 1,000 children), New Jersey’s prevalence estimate of **29.3** per 1,000 children was significantly greater than that from any other site, and four sites (Georgia, Maryland, Minnesota, and North Carolina) reported prevalence estimates that were significantly greater than those from any of the five sites in the 13.1–14.1 per 1,000 range.”

