# *Erratum:* Vol. 67, No. SS-6

**DOI:** 10.15585/mmwr.mm6745a8

**Published:** 2018-11-16

In the Surveillance Summary “Prevalence of Autism Spectrum Disorder Among Children Aged 8 Years — Autism Developmental Disabilities Monitoring Network, 11 Sites, United States, 2014,” on page 3, the first sentence of the first full paragraph should have read “ADDM estimates of ASD prevalence among children aged 8 years in multiple U.S. communities have increased from approximately one in 150 children during 2000–2002 to **approximately** one in 68 during 2010–2012, more than doubling during this period (*6–11*).”

